# Cancer neurology in clinical practice, neurological complications of cancer and its treatment (2nd Edn)

**DOI:** 10.1038/sj.bjc.6604960

**Published:** 2009-03-17

**Authors:** I Radovanovic, G Zadeh

**Affiliations:** 1 Division of Neurosurgery, Toronto Western Hospital, University of Toronto, Toronto, Canada

Santosh Kesari, David Schiff and Patrick Wen have released the second edition of their book ‘cancer neurology in clinical practice’. The book addresses the neurological impact of cancer and cancer treatment. Neurological implications of cancers of the central and peripheral nervous system, as well as cancers of other systems are described in well-written and well-organised chapters. The scope of this book is indeed unique as it offers a comprehensive view on topics forming a common denominator to both neurology and oncology, but that are too often neglected in classical textbooks of either disciplines. However, as neuro-oncology is becoming a prominent and true multidisciplinary specialty, there is a need for such a multidisciplinary book addressing practical issues that are encountered every day in the clinical oncological practice. Moreover, as a high number of oncology patients with cancers outside of the nervous system develop neurological symptoms such as pain, headaches, weakness, cognitive disturbances or paraneoplastic syndromes, even the medical oncologist not dealing primarily with brain tumours will quite often be confronted with neurological problems in his or her patients.

The general organisation of the book is logical and facilitates its practical and everyday use. In that regard, the section addressing neurological symptoms such as seizures, the use of corticosteroids, headaches, cognitive problems and pain is particularly useful for clinical practice. The chapter on cognitive dysfunction and mood disorder is particularly useful and important, as it fully recognises and stresses the importance of cognitive decline, loss of quality of life and depression in neuro-oncological patients in general, be it from direct tumoural progression, therapeutical intervention by radiotherapy, chemotherapy or a combination of both.

The specific chapters addressing less frequent but equally important, problems in oncology such as dural metastasis, vascular complications of cancer and paraneoplastic disease are one of the main strengths of this textbook, as these subjects are often given less attention in textbooks. Individual chapters may present some redundancies; however, this is because of the overlap in many general processes such as pain, paraneoplastic disease or complications of chemotherapy.

Overall this textbook is very comprehensive and encompasses main neuro-oncological challenges. In our opinion there are few minor and emerging aspects of cancer neurology that are not touched upon. For example, the chapter on pain and the chapter on metastasis miss the emerging role of vertebroplasty for the management of spine metastasis and metastatic spine fractures, which has proven to have a very beneficial effect on vertebral metastatic pain. The chapter on neurological complications of systemic paediatric cancer is very welcome, as the management of this specific patient group represents a specific challenge. However, a chapter or a chapter section on primary paediatric brain tumours, important specific issues such as craniospinal radiation and neurological impact of tumours of the posterior fossa in children would be useful.

In conclusion Schiff, Kesari and Wen have edited a very elegant and highly practical textbook, written by recognised authorities in their respective fields, which will be used by a wide range of medical and surgical specialists who are confronted on a daily basis with neurological manifestations of cancer in their practice.

